# Annotations

**Published:** 1895-08-24

**Authors:** 


### Annotations.

### "Jerry-built" Houses and Diphtheria.

The increase of diphtheria in large towns, more especially in the southern and eastern parts of England, has been so alarming during the last eight or ten years that every medical or sanitary congress has taken to discussing the subject with a view to the discovery of the cause. For some time public elementary schools have borne the brunt of the blame; but at the British Institute Congress the other day Professor W. R. Smith showed reasons for believing that such schools are not the real founts of origin of the numerous epidemics which have prevailed. One speaker made a suggestion which may prove to be important. He affirmed his conviction that "jerry-built" houses contained stored-up poison germs, which, being liberated under conditions of extreme concentration, might give rise to sporadic cases of diphtheria. If such houses are built on small and unwholesome areas, it is easy to see that extensive epidemics may thus originate. That diphtheria germs may be present in the vile refuse which often goes to the erection of "jerry-built" houses, and that they may develope under the conditions of heat and concentration with which they are then associated, we can well believe. The whole subject of "jerry building" is fraught with danger to the public, and demands the most searching investigation on the part of our highest medical and sanitary authorities.

### The Doctor's Verdict.

To the patient how important is the verdict! And yet to the doctor how grave is often the doubt how far he is justified in disclosing what is passing in his mind! It is, indeed, an anxious matter to decide how far a medical man should take a patient into his confidence in regard to the details and especially in regard to the prospects of his case. For the doctor's own protection there can be but little question that it is often the wisest course to tell the patient everything. For the patient's sake, however, this is not always the case. Not that we would ever either advise or even sanction the telling of an untruth. Even putting morality on one side, and considering candour as a mere matter of treatment, it may at once be said that nothing is worse for a patient than to lose confidence in the honesty of his medical attendant. But it often happens that a case is involved in doubt; not the sort of doubt which arises from obscurity in diagnosis, or can be cleared up by a consultation, but doubt as to its future progress, a matter which can only be solved by time; and while we feel assured that when a medical man is asked to tell the truth about a case he ought to deal honestly and give truth so far as knowledge goes, we are equally assured that it is neither kind nor right that he should make his patient a sharer in his doubts. The mind of a sick man is not able to judge questions of this sort fairly, his ignorance of terms makes it impossible for him to understand the true bearing of the facts, his personal interest is far too intense to make it possible for him to form a just estimate of the argument. We repeat, then, the doctor should, if asked for it, state his opinion, after giving the best powers of his mind to the forming of a true and just one; but it is no part of his duty to state the grounds on which that opinion is founded, or to lay bare before the patient all the doubts and difficulties, all the frightful eventualities which may perchance present themselves—things which, when anxious cases are on hand often destroy all rest and peace of mind, and for a time make the conscientious doctor's life a nightmare. It is, doubtless, hard that, in addition to deciding on the treatment of a case, a doctor should be asked to tie himself down to a prognosis. But if he is so asked, his first duty is either to make up his mind or say he does not know. He has no right either to prop up his patient with false hopes, or to pour out before him all the possibilities of the case, leaving the sick man to worry over a problem on which the doctor, with all his education, can come to no conclusion.

### The Tottenham Hospital Dispute.

The committee appointed to take evidence on the various matters which have of late been in dispute at the Tottenham Hospital has closed its sittings and issued its report. The members of the committee, it may be remembered, were Mr. H. M. Bompas, Q.C., Mr. Pearce Gould, F.R.C.S., and Dr. Biss. It is much to be regretted that the report, which is couched in sober and non-contentious terms, should be so singularly disappointing from a practical point of view. In every important particular it leaves things exactly as they were before. It is understood that the leading members of the medical profession in Tottenham, who have from the first taken up a very dignified and impartial position, intend to issue, as soon as may be practicable, what they hold to be a correct statement of the actual facts, and certain recommendations of a practical kind which they consider that the occasion demands. We await the publication of the "statement" before arriving at a final conclusion. In the meantime two points may be referred to, both of which have been dealt with by Mr. Bompas's committee. The first point has reference to the "director" of the hospital, Mr. Newton. The committee state that the director "is comparatively young, and think it would be to the advantage of the institution that his place should be filled by a married man of greater age and experience." But, they go on to suggest that Mr. Newton shall be appointed secretary instead of director. Now in an institution like a hospital a "secretary" is a "director" and a "director" is a "secretary." In other words, the committee advise the removal of Mr. Newton because he is unsuitable, in one breath, and in the very next advise his retention under another name. The second point has reference to the lady superintendent. The committee affirm that by defect of training and experience she is not competent to administer the department of the hospital's work which deals with the nursing of the patients and the instruction of nurses. Nevertheless, they propose to make the new head of the nursing department subordinate to the present lady superintendent! Enough, we think, has been said to show that the dispute at Tottenham can by no means be considered at an end.

